# Artificial intelligence in pharmacovigilance: a narrative review and practical experience with an expert-defined Bayesian network tool

**DOI:** 10.1007/s11096-025-01975-3

**Published:** 2025-07-30

**Authors:** Rogério Caixinha Algarvio, Jaime Conceição, Pedro Pereira Rodrigues, Inês Ribeiro, Renato Ferreira-da-Silva

**Affiliations:** 1 Faculty of Sciences and Technology, University of Algarve, Faro, Portugal; 2 Algarve Biomedical Centre Research Institute (ABC-Ri), University of Algarve, Faro, Portugal; 3 Centre for Interdisciplinary Studies (CEIS20), University of Coimbra, Coimbra, Portugal; 4 RISE-Health, Department of Community Medicine, Information and Health Decision Sciences, Faculty of Medicine of the University of Porto, Porto, Portugal; 5 Porto Pharmacovigilance Centre, Faculty of Medicine of the University of Porto, Alameda Prof. Hernâni Monteiro, 4200-319 Porto, Portugal

**Keywords:** Artificial intelligence, Drug-related side effects and adverse reactions, Machine learning data mining, Natural language processing, Pharmacovigilance

## Abstract

**Background:**

Pharmacovigilance is vital for monitoring adverse drug reactions (ADRs) and ensuring drug safety. Traditional methods are slow and inconsistent, but artificial intelligence (AI), through automation and advanced analytics, improves efficiency and accuracy in managing increasing data complexity.

**Aim:**

To explore AI’s practical applications in pharmacovigilance, focusing on efficiency, process acceleration, and task automation. It also examines the use of an expert-defined Bayesian network for causality assessment in a Pharmacovigilance Centre, demonstrating its impact on decision-making.

**Method:**

A comprehensive literature narrative review was conducted in MEDLINE (via PubMed), Scopus, and Web of Science using a set of targeted keywords, including but not limited to “pharmacovigilance”, “artificial intelligence”, “adverse drug reactions” and “drug safety”. Relevant studies were analysed without restrictions on publication year or language. The search was carried out in January 2025.

**Results:**

AI has greatly improved pharmacovigilance by streamlining signal detection, surveillance, and ADR reporting automation. Techniques like data mining and automated signal detection have expedited safety signal identification, while duplicate detection has enhanced data precision in safety evaluations. AI has also refined real-world evidence analysis, deepening drug safety and efficacy insights. Predictive models now anticipate ADRs and drug-drug interactions, enabling proactive patient care. At a regional pharmacovigilance center, the implementation of an expert-defined Bayesian network has optimized causality assessment, reducing processing times from days to hours, minimizing subjectivity, and improving the reliability of drug safety evaluations.

**Conclusion:**

AI holds significant promise for enhancing pharmacovigilance practices, yet its practical application remains primarily confined to academic research, with integration hindered by data quality issues, regulatory barriers, and the need for more transparent algorithms.

## Impact statements

- Artificial intelligence (AI) is enhancing pharmacovigilance by automating adverse drug reaction (ADR) detection, improving signal identification, and enabling real-time risk assessment through advanced data processing.
- AI models can uncover hidden patterns in pharmacovigilance datasets, allowing earlier signal detection and faster safety interventions than traditional methods.
- Despite their promise, AI tools face challenges related to data quality, regulatory acceptance, and algorithm transparency, reinforcing the need for robust validation and explainable outputs.
- Bridging the gap between AI potential and routine pharmacovigilance practice requires standardised frameworks, regulatory alignment, and trust in automated decision support systems.
- Expert-defined Bayesian networks improve causality assessment by reducing subjectivity, increasing consistency, and enabling more transparent, data-driven decisions.
- In a real-world pharmacovigilance centre, the implementation of a Bayesian network significantly reduced case processing times while maintaining high concordance with expert judgement.

## Introduction

Pharmacovigilance encompasses the detection, assessment, understanding, and prevention of adverse drug reactions (ADRs) or other drug-related safety issues [1]. It plays a vital role in public health by ensuring the continued safety of medicines after marketing authorisation and enabling timely interventions to mitigate risks. ADRs are estimated to account for 2.7–15.7% of hospital admissions and occur in approximately 17% of hospitalised patients [2].

Despite its importance, pharmacovigilance faces increasing challenges in adapting to the evolving healthcare landscape. Traditionally, it has relied on spontaneous reporting systems and expert-driven manual assessments. While these methods have long been the cornerstone of drug safety surveillance, they are proving increasingly inadequate due to the exponential growth in pharmacological and clinical data [3]. The digital transformation of healthcare, particularly the integration of electronic health records (EHRs) and the proliferation of big data sources, has dramatically expanded the scale and heterogeneity of information available for safety monitoring [3–5]. This is especially relevant in precision medicine, where personalised therapies produce intricate and highly individualised datasets [6]. Furthermore, much of the data is unstructured and scattered across multiple systems, limiting its usability for pharmacovigilance through conventional analytical methods [7].

AI is transforming the analysis of unstructured data, such as spontaneous reports and social media posts, which contain valuable yet difficult-to-process information [8, 9]. This capability accelerates safety signal detection and enhances responses to emerging threats [10, 11], highlighting the need for innovative analytical approaches to improve ADR detection and management [12, 13]. AI applications in pharmacovigilance range from automating routine tasks to addressing complex analytical challenges, with techniques such as machine learning (ML), deep learning (DL), and natural language processing (NLP) enabling the efficient analysis of vast amounts of structured and unstructured health data [14]. Among AI methodologies, Bayesian networks (BNs) stand out for modelling complex probabilistic relationships under uncertainty, using directed acyclic graphs to capture conditional dependencies and predict adverse outcomes from observed data [15]. BNs are particularly valuable in pharmacovigilance because they can incorporate prior knowledge and learn from data simultaneously, enabling the integration of diverse sources such as spontaneous reports, EHRs, and genomic data [16]. Their transparent structure also facilitates the interpretability of results, which is crucial for regulatory decision-making and clinical validation [17]. As a result, BNs are increasingly explored for tasks such as ADR signal detection, causality assessment, and risk stratification in pharmacovigilance systems [18, 19].

These methodologies enhance risk–benefit assessments and uncover hidden patterns and associations that might go unnoticed, strengthening pharmacoepidemiological studies and monitoring drug safety. Recent public health crises, such as the COVID-19 pandemic, and the introduction of accelerated drug approvals have underscored the need for rapid post-marketing safety data generation [5]. Moreover, Yang et al*.* [20] demonstrated that a DL model applied to free-text hospital ADR reports improved allergic reaction detection by 24% and reduced the need for manual review by 64%, reinforcing AI’s potential to optimize pharmacovigilance workflows. Although not yet a reality in routine pharmacovigilance, AI shows great potential in detecting ADRs and Drug–Drug Interactions (DDIs) by processing unstructured data in real-time. It improves safety signal detection and causality assessments, uncovering drug-ADR links missed by traditional methods [4, 21, 22]. AI also enhances Real-World Evidence (RWE) integration, offering a comprehensive view of drug safety for diverse populations, making it essential for proactive surveillance and post-marketing evaluation.

The prediction of ADR in susceptible patients is another example where AI has shown great potential. ML models can be trained to identify patients at higher risk of developing an ADR based on their genetic profiles and medical histories [23]. Hu et al. [9] tested several ML models to predict ADRs in older inpatients. Among the various models, one stood out with the highest performance, achieving an accuracy of 88.06%. The study highlighted key risk factors such as the number of drugs, age, and medical conditions, reinforcing the value of AI in enhancing patient safety by predicting ADRs in high-risk populations.

Nevertheless, despite AI's promises, its pharmacovigilance implementation is not without challenges. Integrating these tools into regulatory systems requires standardization of data and protocols, as well as rigorous validation to ensure that decisions made by AI algorithms are transparent, explainable, and reliable [4, 22].

### Aim

To explore the practical applications of AI in pharmacovigilance, highlighting its added value in efficiency, process acceleration, and task automation. Furthermore, it will present an example of the routine use of an expert-defined Bayesian network for causality assessment in a Pharmacovigilance Centre, illustrating the potential of AI technologies in supporting informed decision-making processes.

## Method

A narrative review methodological approach was adopted to critically synthesise the application of AI in pharmacovigilance, while maintaining flexibility to incorporate emerging regulatory and practical considerations.

### Search strategy and data sources

A comprehensive literature search was conducted in MEDLINE (via PubMed), Scopus, and Web of Science in January 2025. Both MeSH terms and free-text keywords were used, including: pharmacovigilance, artificial intelligence, machine learning, natural language processing, drug-related side effects and adverse reactions, data mining, electronic health records, big data, clinical decision support systems, Bayesian networks, deep learning, drug safety, signal detection, and spontaneous reporting systems. Boolean operators (AND/OR) were applied to refine the search. Reference lists of key articles were manually screened (snowballing) to identify additional relevant publications. No restrictions were applied regarding publication date or language.

### Study selection and eligibility criteria

Studies were selected based on their relevance to AI applications in pharmacovigilance, focusing on methodological approaches, practical implementation, and regulatory or policy implications. Inclusion criteria encompassed empirical studies (quantitative or qualitative), methodological papers, and policy or regulatory analyses directly addressing the use of AI in pharmacovigilance or drug safety monitoring. Empirical studies were prioritised, particularly those addressing ADR detection, signal identification, causality assessment, and risk prediction. Exclusion criteria comprised pre-clinical studies, animal studies, opinion pieces lacking technical or regulatory analysis, and articles not directly addressing pharmacovigilance or drug safety contexts.

### Screening and data extraction

Two reviewers independently screened titles and abstracts for eligibility, followed by full-text assessment of potentially relevant articles. Discrepancies were resolved through discussion and consensus. Data extraction was performed thematically, capturing details on AI techniques used, types of data sources analysed (e.g., spontaneous reporting systems, electronic health records), validation methods applied, practical applications within pharmacovigilance workflows, and the reported or potential impact on regulatory and clinical practice.

### Data synthesis

Key concepts and insights were identified and grouped in alignment with the review objectives to facilitate a structured narrative synthesis. The synthesis focused on illustrating how AI methodologies are currently applied within pharmacovigilance systems, their performance and validation approaches, and barriers and opportunities for integration into regulatory frameworks.

Finally, a descriptive illustration of a real-world implementation of an expert-defined Bayesian network within a pharmacovigilance centre was included to contextualise practical application, exemplifying the potential of AI frameworks to support ADR signal detection and prioritisation in operational settings.

## Results

AI is revolutionizing pharmacovigilance by addressing critical challenges in managing, analyzing, and interpreting vast and complex datasets [16, 24]. In the following sections, the main contributions of AI to pharmacovigilance are presented according to thematic areas of application, highlighting its potential to automate processes, enhance risk prediction, and strengthen drug safety monitoring.

### AI in signal detection & pharmacovigilance automation

A foundational step in pharmacovigilance automation is the detection of duplicate reports. Ensuring the integrity and uniqueness of individual case safety reports is essential before advancing to broader tasks such as safety surveillance and signal detection. Duplicate entries can distort safety analyses and lead to misleading conclusions about a medicine’s risk profile. To address this, the Uppsala Monitoring Centre (UMC) developed the vigiMatch algorithm, which applies ML techniques to identify potential duplicates by analysing similarities in patient demographics, drug information, and adverse event descriptions [25]. UMC has applied NLP techniques to analyze unstructured ADR reports, offering a deeper understanding of their content and identifying duplicates that traditional methods might overlook [26]. By integrating AI-driven duplicate detection, pharmacovigilance systems improve data quality, enhance confidence in detected safety signals, and support more informed decision-making, ultimately benefiting public health [27, 28].

Following the elimination of duplicate reports, AI plays a central role in expanding pharmacovigilance automation. It enables the proactive identification of safety signals through ML algorithms and predictive analytics, improving risk management and patient safety [8, 29]. AI supports automated surveillance systems capable of processing large-scale datasets continuously and efficiently [30, 31]. Real-time data analysis further contributes to the early detection of safety trends, enhancing the responsiveness of pharmacovigilance activities.

A key component of these advances is data mining, which allows the analysis of massive and heterogeneous datasets to identify potential safety signals. These may include both structured and unstructured sources, such as ADR reports, EHRs, and social media platforms [10]. Computational methods have improved the capacity to detect ADRs earlier in the product lifecycle by revealing associations that are not easily detected through traditional approaches.

Data mining techniques such as neural networks, decision trees, and clustering algorithms are applied to classify and detect relationships between drugs and ADRs. Harpaz et al. demonstrated that such methods improve signal detection rates in large pharmacovigilance databases [8, 16]. Additionally, text mining plays a key role in analysing the narrative sections of ADR reports. Studies by Botsis et al. and others show that these techniques uncover patterns and linguistic cues often missed in manual review [32, 33]. Together, these AI-driven tools enhance the sensitivity and precision of pharmacovigilance systems, supporting a shift from reactive to anticipatory models of drug safety surveillance. Moreover, DL approaches have also emerged as powerful tools in this space. As highlighted by Chen et al., DL is particularly effective in handling large datasets with complex, non-linear relationships [34, 35]. Techniques like association rule mining and Bayesian frameworks have further proven helpful in identifying unexpected links between drugs and ADRs, especially in spontaneous reporting systems [36]. Convolutional neural networks (CNNs) improve detection accuracy when analyzing textual and visual data, while Recurrent Neural Networks provide insights into the temporal progression of drug safety signals using sequential datasets such as timestamped ADR reports [37, 38]. ML models also support predictive pharmacovigilance by identifying novel patterns in historical data, allowing for proactive ADR prevention [11]. Hybrid models that combine traditional disproportionality analysis with ML algorithms are increasingly being adopted to enhance signal detection by improving sensitivity and specificity without compromising interpretability [39].

The integration of AI in pharmacovigilance is also evident in automated signal detection systems. Unlike conventional retrospective approaches, these models continuously monitor incoming data from sources like EHRs, spontaneous reports, and social media [24, 40]. Key methods include NLP, clustering, and supervised learning, which allow for the real-time identification of high-priority safety signals [41, 42]. This proactive strategy contrasts with traditional methods that rely on manual data exploration or predefined queries [43]. Automated systems not only accelerate ADR detection but also enhance reliability. By analyzing data in real time, they facilitate timely risk identification and help healthcare professionals focus on interpretation and decision-making rather than laborious data review [44]. Coste et al. emphasized that such systems outperform manual techniques in both speed and scope [24] emphasized that such systems outperform manual techniques in both speed and scope [24]. Furthermore, automated tools offer a more comprehensive view of drug safety by integrating diverse data sources like reporting systems, EHRs, and patient-reported outcomes [45].

The role of AI in continuous drug monitoring is increasingly vital for patient safety. NLP, for instance, accelerates data extraction from free-text ADRs, as shown by Hu et al. [9]. Meanwhile, reinforcement learning (RL) models are gaining traction for their ability to refine detection algorithms based on feedback, making them more relevant to real-world scenarios [8]. As these technologies evolve, the incorporation of explainable AI (XAI) will be essential to ensure transparency, accountability, and regulatory compliance in pharmacovigilance systems [46].

### Data integration and evidence generation

Understanding the safety and effectiveness of medications after they are marketed requires integrating data and studying RWE. The challenge is in efficiently combining and evaluating the growing amount of data provided from real-world sources, such as ADR reports, insurance data, and EHR. AI makes the integration of complex and heterogeneous data easier, which helps derive valuable insights for pharmacovigilance [47].

#### Multidimensional data integration

AI integrates diverse data sources, including structured and unstructured formats like medical images, clinical notes, and test results. ML and NLP techniques enable a more contextualized understanding of adverse events [48]. Additionally, AI-driven harmonization standardizes heterogeneous datasets, improving interoperability. Algorithms align terminologies from various sources, such as SNOMED CT and MedDRA, enhancing consistency and cross-referencing [49].

#### Real-world evidence analysis

When it comes to understanding how medications are used outside of the controlled setting of clinical trials, RWE analysis offers important information. AI makes it easier to analyze massive amounts of RWE by pointing out links and patterns that conventional analyses could miss [50]. For example, AI expedites the identification of rare or long-term harmful effects by automating safety signal detection in real-time [51]. RWE analysis is essential for this purpose. Additionally, applying AI to RWE makes it possible to detect differential safety signals and effectiveness patterns across subpopulations, such as age groups or comorbidity profiles, supporting a more individualised pharmacovigilance strategy [51].

A key advancement in RWE analysis with AI is federated learning, which enables collaborative data analysis across institutions without sharing sensitive patient information [52]. This method preserves privacy while incorporating diverse datasets, enhancing the generalizability of safety insights [53].

However, issues like inconsistent data quality and the need for transparency in AI models remain. AI is valuable for data integration and RWE analysis, processing large datasets quickly and identifying complex patterns [54].

### Predictive models for adverse drug reactions and drug-drug interactions

Predictive models are revolutionizing pharmacovigilance by enabling early detection of ADRs and DDIs. These models, powered by ML algorithms, analyze large-scale datasets, including spontaneous reports, EHRs, and clinical trials [16]. Identifying patterns in patient demographics, genetic data, and pharmacological properties, provides a more proactive and precise risk assessment, which is particularly relevant given the increasing prevalence of polypharmacy, especially in older populations [55]. 

Different ML techniques have unique strengths for ADR and DDI prediction. Random forests are effective in identifying ADR risk factors like drug-induced liver injury [56]. DL and neural networks detect complex DDIs by analyzing molecular structures and biological interactions [57]. RNNs excel in processing sequential data, making them valuable for detecting temporal ADR patterns in longitudinal patient records [58, 59].

Algorithm selection depends on data type. Random forests excel with structured datasets, while DL models are better for unstructured data, like genetic sequences or medical images [60]. Decision trees and support vector machines are often used to classify ADR risks based on drug properties and patient variables [61], as shown in Table 1.

**Table 1:** Common machine learning algorithms for predicting adverse drug reactions and drug-drug interactions

Algorithm	Application	Strengths	Limitations
Random Forest	Early detection of ADRs (e.g., drug-induced liver injury)	High accuracy; can handle missing data	Computationally intensive; prone to overfitting
Decision Trees	Predicting ADRs based on patient data and drug properties	Easy to interpret; fast execution	Less effective with noisy data
Neural Networks	DDIs prediction through pattern recognition	High flexibility; can model complex relationships	Requires large datasets; less interpretable
Support Vector Machines	Classifying ADR risk based on structured data	Effective for high-dimensional datasets	Difficult to tune; sensitive to parameter choices
Deep Learning	DDI prediction using molecular and biological data	High accuracy; capable of modeling intricate relationships	Requires extensive data; computationally expensive

*ADRs* Adverse drug reactions; *DDI* Drug-drug interactions

Predictive models assess risks in real-time, often before clinical symptoms appear, helping healthcare professionals make informed prescribing decisions, especially in complex cases with multiple medications [62]. These models can complement post-marketing surveillance, improving patient outcomes and reducing serious ADR occurrences [63]. An illustrative example is the use of AI-based models to predict the likelihood that a reported case will be classified as serious. Although regulatory definitions of seriousness exist, in practice, many reporters rely on personal perception or patient-reported impact rather than formal criteria. Predictive models that identify key clinical or sociodemographic variables associated with case seriousness can support pharmacovigilance centres in validating reports and prioritising follow-up [64].

Predictive models face challenges, with accuracy relying on the quality and diversity of input data [65]. Biases in datasets can lead to flawed predictions, threatening patient safety if not validated. The "black-box" nature of ML algorithms, especially DL models, raises concerns about transparency and interpretability [66], key for clinical decision-making (Table 2).

**Table 2:** Benefits and challenges of artificial intelligence-driven predictive models for adverse drug reactions and drug-drug interactions

Domain	Benefits	Challenges
Accuracy	Identify patterns not visible to human analysts	Dependent on data quality and diversity
Speed	Automates ADRs and DDIs detection in real-time	Requires significant computational resources
Scalability	Can handle large datasets with multiple variables	Potential bias in models, especially if data is incomplete
Personalization	Supports personalized medicine approaches	Difficult to interpret outputs from black-box models
Validation	Reduces reliance on post-marketing surveillance alone	Continuous validation needed to maintain accuracy

*ADRs* Adverse drug reactions; *DDI* Drug-drug interactions

Predictive models will develop to incorporate new data types, such as proteomics, metabolomics, and genomes, as healthcare data volume and complexity continue to rise [67]. This will allow for the provision of individualized ADR and DDI forecasts. Big data analytics and AI integration can further improve pharmacovigilance by enabling precision medicine strategies that account for patient variability when evaluating drug risks [68]. Furthermore, XAI approaches are anticipated to enhance model transparency, boosting patient and clinician confidence [69].

### Causality assessment: from traditional to AI-driven approaches

In pharmacovigilance, causality evaluation links drug administration to adverse events. Traditionally, expert judgment and the clinical differential diagnosis method, using Bradford Hill criteria, guide this process. Standard tools like the Naranjo algorithm and Bayesian inference-based methods provide semi-quantitative or qualitative support. However, these methods are time-consuming, variable between evaluators, and depend on trained professionals expertise [70, 71].

The advent of AI has transformed causality evaluation by introducing systems capable of analyzing vast and heterogeneous datasets with speed and precision. AI-based approaches, such as ML models, are designed to incorporate probabilistic reasoning, operational algorithms, and even expert-driven methodologies like introspection. For instance, NLP techniques can extract critical information from unstructured data, such as patient narratives and clinical notes, while neural networks uncover patterns in complex datasets that may not be evident through traditional methods [72]. These advancements reduce the cognitive burden on human experts, enhance objectivity, and facilitate the integration of RWE for more robust and scalable causality assessments. Importantly, XAI ensures transparency in these automated processes, enabling regulators and clinicians to trust the outputs and incorporate them into decision-making frameworks (Table 3) [73].

**Table 3:** Summary of tools, benefits, challenges, and the role of experts in causality assessment with artificial intelligence [3, 7, 25]

Automated tools	AI technologies can swiftly identify causal linkages by using algorithms like the Naranjo algorithm and Bayesian approaches. These instruments analyze massive amounts of data and spot subtle patterns
Benefits of AI	AI lessens human bias, handles massive amounts of data effectively, and advances constantly through machine learning. Adverse event assessments are now faster and more accurate thanks to these advancements
Challenges of AI	The interpretation of algorithmic judgments, stringent model validation, and the requirement for transparency to win over healthcare professionals trust are among the challenges
The role of experts in the era of AI	Although AI offers data-driven recommendations, results must be interpreted in context, requiring the knowledge and clinical judgment of specialists. Human-AI collaboration is essential for making good decisions

*AI* Artificial intelligence

### Expert-defined Bayesian network in a pharmacovigilance centre

Causality attribution in ADR reporting is a critical component of the pharmacovigilance process, as it determines the likelihood that a specific drug is responsible for a reported adverse event. While various methods exist for this evaluation, the Portuguese Pharmacovigilance System employs the Global Introspection method [71, 74]. In this approach, clinical experts assess the probability of a causal relationship between a drug and an ADR based on criteria recommended by the World Health Organization (WHO). These assessments assign degrees of probability, typically ranging from “certain” to “unlikely,” reflecting the confidence level in the drug-reaction association [75]. 

**Fig. 1:**
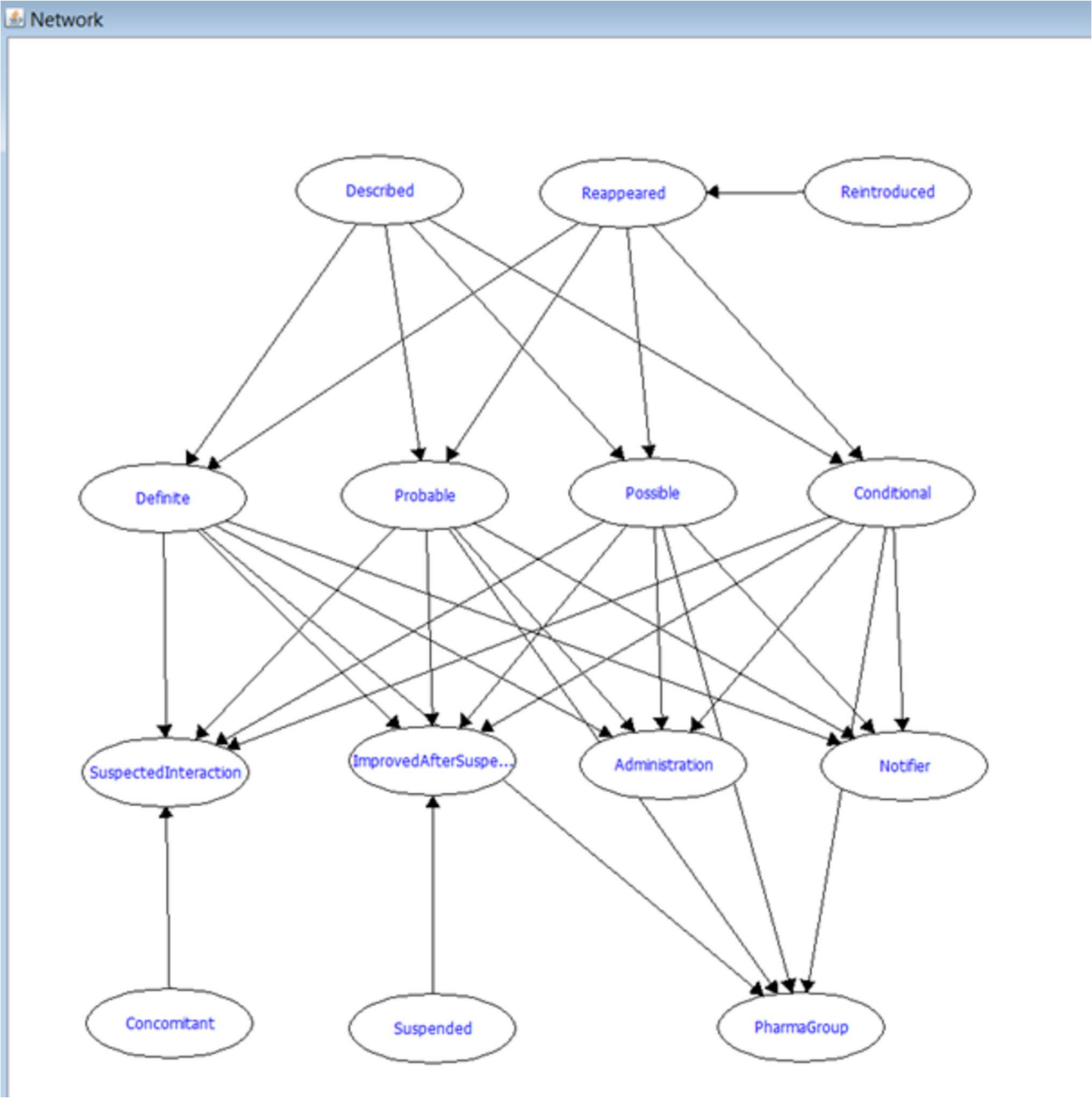
Graphical representation of the expert-defined Bayesian network implemented in a Pharmacovigilance Centre

At the Porto Pharmacovigilance Centre (UFPorto, in the Portuguese acronym), one of the regional pharmacovigilance centers within the Portuguese Pharmacovigilance System, this task is carried out by a multidisciplinary team of pharmacists and physicians. Their expertise is essential for interpreting intricate clinical data, accounting for confounding factors, and evaluating alternative explanations for adverse reactions, ensuring robust and reliable causality assessments. To enhance efficiency and ensure continuity in ADR reporting, especially during periods of limited expert availability, UFPorto implemented an AI system in 2018 based on a Bayesian network [76]. This system acts as a “proxy” for global introspection, emulating the reasoning processes of clinical evaluators. A Bayesian network is a probabilistic graphical model that represents variables and their conditional dependencies through a directed acyclic graph (Fig. 1). In the context of pharmacovigilance, it models the relationships between various factors in ADR reports, such as the suspected drug, dechallenge/rechallenge information, and the characteristics of the ADR [76].

Leveraging data from ADR reports and expert assessments spanning over 12 years at UFPorto, the AI system generates probability scores that simulate how a human expert might assess each case. By automating this process, the system ensures consistent causality assessments and supports timely reporting during periods of high workload or when clinical experts are unavailable. This innovative approach demonstrates how AI can complement traditional pharmacovigilance methods, maintaining the reliability of causality assessments while improving operational efficiency.

The implementation of the Bayesian network system at the UFPorto has introduced several advantages, significantly enhancing the efficiency and effectiveness of causality assessments. One of the primary benefits is its ability to ensure that these evaluations are completed within regulatory timelines, even during periods of limited human resources. By automating key aspects of the process, the system maintains the centre’s capacity to meet its obligations while alleviating the workload on clinical experts.

Although the Bayesian network system does not replace the expertise of human evaluators, it serves as a valuable tool for providing preliminary assessments, supporting decision-making, and ensuring the continuity of operations. A particularly noteworthy feature of this system is its ability to deliver highly comparable results to those of human experts. A study conducted by the team demonstrated a high degree of concordance between the AI’s causality attributions and the judgments made by clinical evaluators [76]. The positive predictive value for the highest probability levels was 71.4% (*Definitive* level) and 87.3% (*Probable* level).

The Bayesian network's errors were always "conservative," as whenever the network made a mistake, it assigned the case the probability level immediately below the level assigned by the specialist. These promising outcomes have bolstered the centre’s confidence in the system as a reliable adjunct to human expertise, underscoring its role in maintaining high pharmacovigilance standards.

However, this Bayesian network has an inherent limitation, i.e., it may perpetuate potential biases from the clinical expert (gold standard) since its construction was based on cases assessed by that expert. Additionally, the lack of continuous updates with new cases hinders its ability to evolve and adapt to emerging notifications. Notably, updating the network (which dates back to 2018) with ADR reports received since then would be valuable, particularly those from the 2021 COVID-19 vaccination campaign. This campaign significantly increased the volume of ADR reports across all pharmacovigilance centers, including the UFPorto, and introduced a broader range of reports, including those from consumers (non-health professionals) and reports on vaccines under additional monitoring, which were subject to intense media attention.

Another operational limitation is the network's graphical interface, which is not very user-friendly. Users require prior training to input the necessary data for the network to function and interpret its results. To address this, plans are underway to develop a more intuitive user interface, facilitating the network's dissemination to other national and potentially international pharmacovigilance centers.

### Case study

*The spontaneous report refers to a case submitted by a pharmacist about a male patient, aged 50, who developed urticaria associated with the use of ciprofloxacin 500 mg, with unknown therapeutic indication, with a dosage regimen of one tablet (500 mg) every 12 h. The ADR began on the same day as the administration of the suspected medication (the exact time-to-onset is unknown), lasted for one day, and required the withdrawal of ciprofloxacin and specific treatment with deflazacort. There is a reference to the concomitant use of paracetamol. No history of ADRs to any drug is known. No relevant clinical history is known ADR Outcome: Recovered.*

**Fig. 2:**
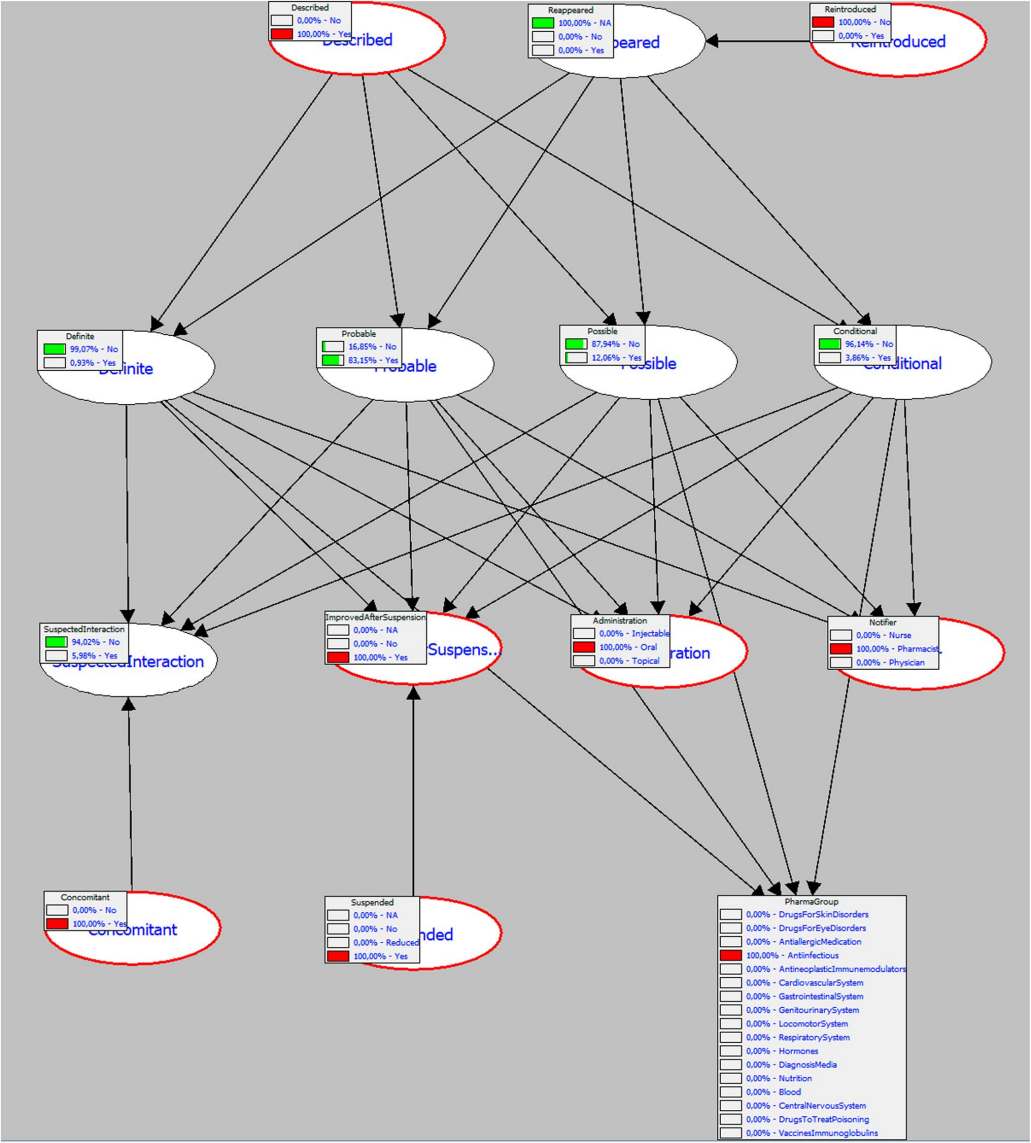
Example of Bayesian network application to a real pharmacovigilance case with probability estimation

The image below illustrates how to assess this case using the Bayesian network, and the interpretation of the result is as follows: “The network has 83.15 per cent confidence that the expert assessor would give this case the grade of *Probable*” (Fig. 2).

The integration of a Bayesian network system at UFPorto has enhanced the centre’s ability to meet regulatory deadlines while maintaining consistency with expert clinical reasoning. As advancements in AI progress, such systems hold significant potential to further streamline pharmacovigilance processes, providing reliable and efficient tools for causality assessment in drug safety monitoring.

## Discussion

Integrating AI in pharmacovigilance offers opportunities to enhance drug safety but also presents challenges, particularly regarding the quality and heterogeneity of data. Much information in spontaneous reporting systems and EHRs is unstructured or incomplete, complicating analysis and limiting insights [77]. Universal data standards, such as those from the International Council for Harmonisation (ICH), and AI-driven tools for data normalization can improve data quality, enabling more accurate signal detection. 

A challenge in pharmacovigilance is the complexity of AI algorithms in tasks like causality assessment and ADR prioritization. While ML models analyze large datasets, their “black box” nature raises concerns about transparency, especially in regulatory decisions and high-risk evaluations. To address these issues and improve the reliability of AI applications in pharmacotherapy, the MedinAI guideline provides structured recommendations to ensure methodological clarity and reporting transparency in AI-based studies applied to pharmaceutical contexts [78]. Algorithms trained on specific populations may not generalize, risking biased conclusions. XAI offers interpretable outputs, fostering trust and validation, while feedback loops help models adapt to evolving data, improving safety signal detection and causality assessments [25]. XAI frameworks are being developed to ensure AI-generated recommendations are interpretable and validated by experts, addressing ethical and regulatory concerns. To ensure accuracy, AI solutions must undergo rigorous testing [79].

Ethical and legal considerations present significant challenges in pharmacovigilance, particularly regarding handling sensitive patient data, which raises concerns about privacy and compliance with regulations like the General Data Protection Regulation [54]. Recent research discusses the ethical and legal considerations associated with the use of AI in clinical pharmacy, emphasizing the need for robust regulatory frameworks to ensure patient safety and efficacy [54, 80]. The high costs associated with developing and maintaining AI systems further limit accessibility to resource-rich centers. Collaborative public–private funding models and global regulatory guidelines could address these barriers by standardizing the validation of AI tools for signal detection, ADR monitoring, and causality evaluation. Addressing the human element is crucial, as AI’s effectiveness relies on professionals who can interpret and apply its outputs. Many pharmacists and physicians lack adequate training, leading to skepticism and underutilization. Targeted education programs and intuitive interfaces adapted to pharmacovigilance workflows can bridge this gap, promoting adoption and trust in AI technologies [81, 82].

AI offers transformative opportunities in pharmacovigilance. By harmonizing databases, AI enables real-time signal detection across regulatory regions, enhancing global drug safety monitoring. NLP helps integrate spontaneous reports by resolving language and reporting inconsistencies. Predictive analytics strengthen early risk detection, while AI frameworks like WHO’s UMC improve collaboration between agencies, optimizing signal validation and standardizing methodologies for better interoperability [27, 30].

Future opportunities in AI-driven pharmacovigilance require targeted actions to address current limitations and support effective implementation. Developing XAI solutions remains essential to ensure transparency and build trust in regulatory and clinical decision-making, particularly for causality assessment and signal prioritisation workflows where opaque algorithms can hinder interpretability. To complement this, improving algorithm adaptability to diverse populations and evolving datasets—for example, through domain adaptation and transfer learning—can help mitigate biases caused by data heterogeneity across pharmacovigilance systems. Tackling financial barriers through collaborative funding models, such as public–private partnerships focused on validating AI models for post-marketing surveillance, can, in turn, make these technological advances accessible in resource-limited contexts. Equally, embedding targeted training and education—including AI modules in pharmacovigilance curricula and user-friendly dashboards within centres—is critical to ensure professionals can interpret and apply AI outputs effectively. Strengthening real-time ADR analysis capabilities, supported by federated learning and secure data-sharing infrastructures, further enhances the early detection of rare and long-term safety signals while safeguarding patient privacy. Finally, these efforts must be reinforced by stronger international and regulatory collaboration through shared AI platforms and frameworks—for example, aligning with WHO PIDM initiatives and ICH E19 guidance—to enable harmonised signal detection methods, data interoperability and collective learning that can sustainably advance global drug safety monitoring. In this context, several strategic priorities are emerging as essential to guide the future of AI-based pharmacovigilance. These include the development and implementation of XAI to ensure transparency and build trust in clinical and regulatory decision-making. It is also crucial to improve the adaptability of algorithms to different populations and evolving datasets to mitigate the risk of bias and maintain performance over time. Enhancing real-time ADR monitoring—especially for rare and long-term signals—through approaches such as federated learning can significantly strengthen early signal detection [83]. Reducing financial barriers via collaborative funding models, expanding targeted training and education for healthcare professionals, and fostering international collaboration through shared AI platforms are equally important to ensure equitable, robust, and globally harmonized pharmacovigilance systems [83, 84].

## Conclusion

This article reflects on the potential of AI to address specific and concrete challenges in pharmacovigilance, both in regulatory and clinical contexts. While AI shows significant promise in enhancing key processes like ADR detection and causality assessment, much of its potential remains within academic models and theoretical frameworks, with limited application in routine pharmacovigilance practices. Further efforts are needed to bridge this gap and fully integrate AI into daily pharmacovigilance operations.
